# Prospect of *in vitro* Bile Fluids Collection in Improving Cell-Based Assay of Liver Function

**DOI:** 10.3389/ftox.2021.657432

**Published:** 2021-06-03

**Authors:** Astia Rizki-Safitri, Fumiya Tokito, Masaki Nishikawa, Minoru Tanaka, Kazuya Maeda, Hiroyuki Kusuhara, Yasuyuki Sakai

**Affiliations:** ^1^Department of Chemical System Engineering, Graduate School of Engineering, The University of Tokyo, Tokyo, Japan; ^2^Laboratory of Stem Cell Regulation, Institute for Quantitative Biosciences (IQB), The University of Tokyo, Tokyo, Japan; ^3^Department of Regenerative Medicine, Research Institute, National Center for Global Health and Medicine (NCGM), Tokyo, Japan; ^4^Laboratory of Molecular Pharmacokinetics, Graduate School of Pharmaceutical Sciences, The University of Tokyo, Tokyo, Japan

**Keywords:** *in vitro*, bile fluids collection, liver culture, liver function analyses, cell-based assay

## Abstract

The liver plays a pivotal role in the clearance of drugs. Reliable assays for liver function are crucial for various metabolism investigation, including toxicity, disease, and pre-clinical testing for drug development. Bile is an aqueous secretion of a functioning liver. Analyses of bile are used to explain drug clearance and related effects and are thus important for toxicology and pharmacokinetic research. Bile fluids collection is extensively performed *in vivo*, whereas this process is rarely reproduced as in the *in vitro* studies. The key to success is the technology involved, which needs to satisfy multiple criteria. To ensure the accuracy of subsequent chemical analyses, certain amounts of bile are needed. Additionally, non-invasive and continuous collections are preferable in view of cell culture. In this review, we summarize recent progress and limitations in the field. We highlight attempts to develop advanced liver cultures for bile fluids collection, including methods to stimulate the secretion of bile *in vitro*. With these strategies, researchers have used a variety of cell sources, extracellular matrix proteins, and growth factors to investigate different cell-culture environments, including three-dimensional spheroids, cocultures, and microfluidic devices. Effective combinations of expertise and technology have the potential to overcome these obstacles to achieve reliable *in vitro* bile assay systems.

## Introduction

The liver is one of the largest glands in the body and is pivotal to various metabolic functions, including blood glucose regulation, protein synthesis, and detoxification. These functions are mainly performed within the hepatocyte parenchymal cells. Hepatocytes are responsible for 40–70% of the xenobiotic liver metabolism (Almazroo et al., 2017). Therefore, analyses of hepatocyte functions can be used for toxicities assay.

Presence of liver diseases or injuries also frequently alters the amount and composition of liver secretions (Luo et al., 2018). A functional liver produces bile, a secretion containing 95% water that dissolves bile acids (BAs), bilirubin, ions, hormones, and other metabolites. BAs are major organic solutes, mainly consisting of cholic acid and chenodeoxycholic acid. BAs and bile are secreted into the canaliculi structure of hepatocytes prior to entering the biliary system in the liver (Boyer, 2013; Chiang, 2013). Although bile formation is a common process in normal livers, it is impaired in cases of cholestatic liver disease (Boyer, 2013). The presence of drugs or liver injuries (Luo et al., 2014; Ni et al., 2016) has been widely reported to alter the amount as well as the composition (Luo et al., 2014) and perturbation of BA in the bile fluids (Rodrigues et al., 2014). Thus, bile is used as a source for enzymatic assays (Cell Biolabs Inc, 2017), profiling (Samer et al., 2013; Luo et al., 2014; Bathena et al., 2015), and uptake-efflux testing (Yang et al., 2017). Many bile tests have employed *in vivo* collection using both non-invasive and invasive procedures. Non-invasive procedures include urine (Griffiths and Sjövall, 2010; Hofmann and Hagey, 2014; Bathena et al., 2015), fecal recovery (Ghibellini et al., 2006; Griffiths and Sjövall, 2010), and Entero® testing (Bloomer et al., 2013). Meanwhile, invasive procedures employ serum collections (Luo et al., 2014), biliary sphincterotomies (Navaneethan et al., 2014), duodenal fluid collection, nasobiliary drainage, and cholecystectomies (Bloomer et al., 2013).

*In vitro* liver research has been subjected to long-term projects for the establishment of standard preclinical assays that are still widely implemented in pharmaceutical studies. The *in vitro* liver model offers time efficient results and is flexible for human tissues (Soldatow et al., 2013). It is simple, controllable (Xu et al., 2014), allows for intensive analyses (Fatehullah et al., 2016), and exhibits accurate dose–response relationships related to drug analyses (Soldatow et al., 2013). However, bile fluids collection and testing is rarely constructed in an *in vitro* model. The low amount of bile yields, as represented by the BA concentrations from recovered culture media (Marion et al., 2012) and auto-toxic conjugated-BA produced in culture (Woolbright et al., 2015, 2016) has hindered its further consideration. In this paper, we discuss the current research that both directly and indirectly addresses liver-functional bile production *in vitro*.

## Bile Collection From *in vitro* Liver Culture

### Characterization of Drug-Induced Liver Toxicity Mechanism Through Biliary Secretion

Elucidation of the biliary excretion process is important because it leads to an understanding of drug-induced liver toxicity. BAs are often used as an index for this purpose and are found in blood samples collected from test subjects. They can be evaluated as a biomarker *in vivo* (Wolenski et al., 2017; Luo et al., 2018; Liu et al., 2020). This biomarker can be used to predict biliary excretion because some drugs inhibit the bile salt export pump (BSEP). BSEP is an excretion transporter of hepatocytes for Bas, which induces perturbations of biliary excretion (Funk et al., 2001; Kemp and Brouwer, 2004; Wolenski et al., 2017). Conversely, the use of an *in vitro* liver culture system could provide a more detailed understanding of the biliary excretion process from a molecular biological perspective. For example, it can better detect the transporters involved in bile excretions and their inhibitions caused by drugs (Funk et al., 2001; Kemp and Brouwer, 2004). However, with *in vitro* liver models, because there are no outlets for biliary metabolites in most cases, biliary metabolites accumulate in the bile canaliculi between adjacent hepatocytes, preventing accurate evaluation of drug-induced hepatotoxicity. Additionally, the lack of outlets for biliary metabolites restricts development of *in vitro* models for studying the effects of enterohepatic circulation of biliary metabolites, which have the potential to amplify drug toxicity.

### Increase in New Drug Development

Because the development of a new drug generally takes more than 10 years and can cost more than USD 1B (Hughes et al., 2011), it is desirable to have a system that could evaluate pharmacokinetics more accurately and at a lower cost. To predict the pharmacokinetics of drugs in the human body, experimental animals (e.g., mice and rats) are often used. The advantages of doing so are two-fold. First, one can understand the pharmacokinetics (i.e., absorption, distribution, metabolism, and excretion) in the whole body and not just at specific locations. Second, one can implement realistic toxicity studies of oral and inhalation exposures (Barré-Sinoussi and Montagutelli, 2015). However, the problem of species differences cannot be overcome. Moreover, there have been some cases where side effects and immune responses that were not seen in non-clinical animal studies were found in clinical studies in humans (van Norman, 2019). Another issue is the cost and labor required for animal breeding, breeders, and proper breeding environments. Conversely, the use of human cells cultured on Petri dishes can solve these issues while elucidating the local mechanism of pharmacological action (Funk et al., 2001; Kemp and Brouwer, 2004). Development of physiologically relevant *in vitro* liver models is desired because the liver plays a central role in drug metabolism. For the accurate prediction of pharmacokinetics, an *in vitro* liver model should be able to distinguish whether the parent's metabolized drugs were excreted into the blood or bile. In these models, biliary metabolites could be collected directly.

### Need for Enhancing Bile Collection *in vitro*

Bile fluids collection *in vitro* has limitations. The amount of fluids collected from the culture medium is suggestively low, as presented by BA concentrations in the bile at <1 μg/L per 10^6^ hepatocyte culture (Einarsson et al., 2000). An enzymatic immunoabsorbent assay generally has a concentration limit of 1–5 μg/L BAs (Cell Biolabs Inc, 2017). Additionally, methods for extracting bile fluids from cultures are also quite limited. Bile extraction from harvested hepatocyte cultures is commonly performed to increase bile yield (Setchell et al., 1997; Ramaiahgari et al., 2014). However, these methods require hepatocyte extraction, often resulting in culture damage and shortening of the culture's age.

To date, liquid chromatography (LC)–mass spectrometry (MS) is widely utilized as an analytical method to quantify solutes in bile fluids. It has a rapid bile profiling and a detection limit of 10 ng/L (Perwaiz et al., 2001; Scherer et al., 2009), showing a higher precision of 5 ng/L for ultraperformance LC-MS (Sarafian et al., 2015). Another common method is to calculate the fraction of hepatocytes with and without the canaliculi network (Boyer, 2013). Both methods hardly allow direct quantification of the solutes secreted into the bile. Appropriate clearance is a preferable method that would provide direct secretion while preventing tissue extraction. It may also prolong the culture age, which is suitable for chronic models.

## Ideal Bile Production and Collection for *in vitro* Liver Toxicity Analyses

**Figure 1:**
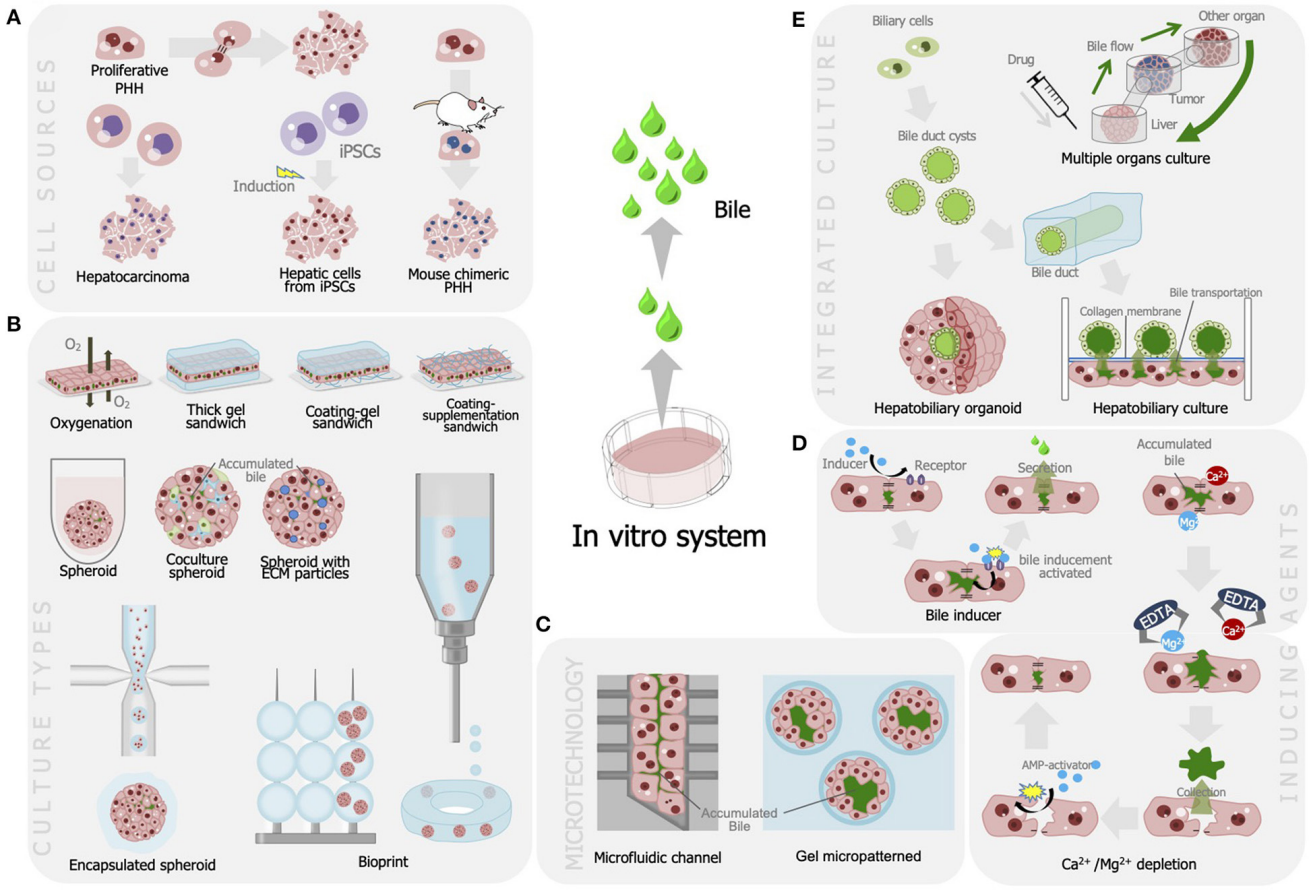
Strategies developed for improving bile production and recovery for liver cell-based assay including **(A)** utilization of various cell sources for optimum bile production; **(B)** modulation of culture model that is efficient for bile secretion and production; **(C)** integration with microfabrication, modulation of bile production, and recovery methods using **(D)** agents and chelates; and **(E)** integration of liver tissues to enable autologous bile recovery.

Numerous studies have been performed to develop advanced bile fluids collection methods and enhance bile assays *in vitro*. These attempts included alterations in the culture model, integration with microtechnology, and bile recovery methods (see Figure 1, Table 1).

**Table 1:** Recent studies that significant on the advancement of bile production and collection from liver *in vitro* model.

**Target of modulation**	**Type of modulation**	**Ideal design for bile production and collection**	**Organism/s**	**Degree of modulation**	**Bile amount collected**	**Relevancy for bile fluids collection**	**References**
Cell source	Long-term primary hepatocytes	OSM-dependent human primary hepatocytes, human hepatocyte-chimeric mice	Human, mouse	Moderate–high	Low–high^a^^,^ ^b^^,^ ^c^	High	Tateno et al., 2004; Nishimura et al., 2005; Azuma et al., 2007; Yamasaki et al., 2010; Levy et al., 2015; Kimura et al., 2019; Ruo et al., 2020
	Hepatocytes cell line	HepaRG		Low	Low–high^a^^,^ ^b^^,^ ^c^	Moderate–High	Andersson et al., 2012; Bachour-El Azzi et al., 2015; Takahashi et al., 2015; Woolbright and Jaeschke, 2015; Woolbright et al., 2015; Susukida et al., 2016
	iPSCs-derived cells	hiHeps		Moderate–High	Low-Moderate^b^	Moderate–Potentially high	Ni et al., 2016; Kvist et al., 2018; Sakai et al., 2019
Culture design	Oxygenated culture	PDMS permeable membrane, Vitrigel membrane	Human, rat	Low^c^^,^ ^b^	Moderate–High	Moderate–High	Matsui et al., 2010; Xiao et al., 2014, 2015; Oshikata-Miyazaki and Takezawa, 2016
	Sandwich culture	Collagen–Matrigel sandwich (thick gel and supplementation)	Human, rat, mouse	Low^a^	Moderate–High	Moderate–High	Swift et al., 2010; Marion et al., 2012; Chatterjee et al., 2014; Fukuda et al., 2014; Keemink et al., 2015; Xiao et al., 2015; Deharde et al., 2016; Lauschke et al., 2016; Ni et al., 2016; Susukida et al., 2016; Watanabe et al., 2016; Ogimura et al., 2017; Yang et al., 2017; Zeigerer et al., 2017; Sun et al., 2019; Ruo et al., 2020
	3D spheroid	Coculture of 3D spheroid, liver organoid, spheroid encapsulation, and bioprinting		Low–Moderate^a^	Moderate–High	Moderate–High	Tamai et al., 2013; Astashkina and Grainger, 2014; Rebelo et al., 2015; Takahashi et al., 2015; Ware et al., 2015; Yamada et al., 2015; Bells et al., 2016; Chan et al., 2016; Lauschke et al., 2016; Ni et al., 2016; Ahmed et al., 2017; Kizawa et al., 2017; Vorrink et al., 2017; Baze et al., 2018; Underhills and Khetani, 2018; Fiorotto et al., 2019
	Micropattern	ECM-Based micropattern		Moderate–High^b^^,^ ^d^	Potentially moderate–High	Moderate–High	Matsui et al., 2012
	Integration with devices	Canaliculi fluidic channel	Rat	High^c^	Potentially moderate–High	Moderate–Potentially high	Nakao et al., 2011; Wang et al., 2018
Transporter activities	Ca^2+^/Mg^2+^ depletion	B-Clear® Technology	Human, rat	Low–Moderate^a^^,^ ^c^	Moderate–High	Moderate–High	Swift et al., 2010; Marion et al., 2012; Fukuda et al., 2014; Bachour-El Azzi et al., 2015; Ni et al., 2016; Yang et al., 2016; Yan et al., 2017; Ying et al., 2018
	Bile salts inducer	Addition of PGE_2_	Mouse	Moderate^c^	Potentially moderate–High	Moderate	Fu et al., 2010; Brouwer et al., 2013
Multi-tissue interactions	Development of bile duct structure	Micropattern cyst-tube making, bile duct differentiation, bile duct *in vitro* morphogenesis	Human, rat, mouse	High^a^^,^ ^c^^,^ ^d^	Potentially high	Potentially high	Tanimizu et al., 2007, 2012; Kido et al., 2015; Sampaziotis et al., 2015; Miura et al., 2018; Rizki-Safitri et al., 2018, 2020; Funfak et al., 2019; Du et al., 2020; Hafiz et al., 2021
	Hepatobilary model	Hepatobiliary spheroid, collagen membrane, ECM-based scaffold	Human, rat	Moderate–High^a^^,^ ^b^^,^ ^c^	Potentially high	Potentially high	Katsuda et al., 2013; Vyas et al., 2018; Wu et al., 2019
	Integrated multiorgans	Liver–intestine model, multiorganoid chip system	Human	High^d^	–^*^	Potentially high	Maschmeyer et al., 2015; Chen et al., 2017; Choe et al., 2017; Skardal et al., 2020
Direct collection of bile		Oil injector	Rat	High^d^	Moderate–High	Potentially high	Matsui et al., 2012

a *Combined with oxygenation.*

b *Combined with organoid culture.*

c *Often combined with sandwich culture.*

d *Combined with micropattern or microfluidics.*

* *Bile salts directly transported and affect the organ of interest*.

### Cell Source for Producing Bile

Primary hepatocytes are the most ideal cell source to represent major liver functions. In both academia and the pharmaceutical industry, the utilization of primary human hepatocytes (PHHs) has been accepted as the gold standard to access human liver function (Hirano et al., 2004; Yamashiro et al., 2006; Maeda and Sugiyama, 2010; Izumi et al., 2017). Donor to donor variations in functions of PHHs can provide valuable information on individual differences in actual human population. However, we should pay attention to some drawbacks to the use of PHHs. In the process of cell preparation, the viability of prepared PHHs is largely dependent on individual batches (Levy et al., 2015; Ruo et al., 2020). Moreover, donor-to-donor variations in the *in vitro* functions of PHHs cannot always correspond to the individual differences in liver functions among actual human population since they come from not only intrinsic hepatic functional variations but other artifacts such as the different situations of cell isolation from donors (e.g., elapsed time from the death of donor to the isolation of PHHs, warm ischemic time, efficiency of collagenase perfusion in the liver) (Olinga et al., 1998; Shitara et al., 2003; Godoy et al., 2013). In academia, the variations could impair the reproducibility and reliability of results. Besides, the cost and limited availability of PHHs sometimes impose a burden on basic research. From this perspective, development of alternative cell sources has been an issue. The establishment of an oncostatin M (OSM)-dependent expansion of PHH-overexpressed human papillomavirus (HPV) oncogenes increases the PHH availability for *in vitro* cultures. It expresses E6 and E7 oncogenes, which are responsible for hepatocyte immortality, as activated by OSM addition. The OSM addition stimulates the proliferation of PHH-overexpressed HPV oncogenes up to 40 populations (doubling), whereas OSM removal results in proliferation and triggers differentiation into mature PHH (Levy et al., 2015). Several groups have reported the generation of chimeric mice with transplantation of human hepatocytes into immunodeficient mice [e.g., urokinase-type plasminogen activator/severe combined immunodeficiency (uPA/SCID) mice (Tateno et al., 2004), Fah^−/−^/Rag2^−/−^/Il2rg^−/−^ mice (Azuma et al., 2007), and TK-NOG mice (Yamasaki et al., 2010)]. In these chimeric mice, large parts of the liver were replaced with transplanted human hepatocytes. The functions of isolated hepatocytes were reported to be comparable with PHHs (Nishimura et al., 2005). Additionally, these chimeric hepatocytes can be maintained with external oxygen supplies, hierarchical cocultures with 3T3 cells, or additions of ECM (Kimura et al., 2019). These studies increase the availability of fresh PHHs instead of cryopreserved cells.

The use of hepatocyte cell lines is also expected to be a suitable alternative to PHH for *in vitro* bile analyses. HepaRG is a bipotent cell line established from hepatocarcinoma that has been extensively utilized for cytochrome P450 (CYP) induction assays and bile analyses (Andersson et al., 2012; Takahashi et al., 2015; Woolbright and Jaeschke, 2015; Woolbright et al., 2015; Susukida et al., 2016). It demonstrates superior BA transport and drug metabolite disposition, as opposed to other common hepatic cell lines (e.g., HepG2) (Takahashi et al., 2015; Woolbright and Jaeschke, 2015; Ni et al., 2016; Susukida et al., 2016; Penman et al., 2019). It also exhibits a similar response to the relevant dose of BA-induced toxicity as PHH (Woolbright et al., 2015). An evaluation of bile metabolites using HepaRG demonstrated that the influx and efflux bile transporters were properly distributed to apical (BSEP, MRP2, MDR1, MDR3) or basolateral (NTCP, MRP3) sites. However, in comparison to PHHs, there are some drawbacks that need to be considered. First, the cost of HepaRG cells per vial is comparable to PHHs. Second, some BA transporter expressions, such as BSEP and NTCP, and the amount of bile secretion in HepaRG were still less compared to that of PHH (Bachour-El Azzi et al., 2015). In addition, some drug-metabolizing enzymes, such as CYP1A2, CYP2A6, and CYP2D6, were reported to have a significantly lower level of expression in HepaRG cells than in PHHs (Andersson et al., 2012). Third, as is true with all cell lines, it is derived from a single donor and thus not suitable to assess the effect of diverse genetic background in actual human population.

Additionally, protocols for liver-cell differentiation have been widely established (Si-Tayeb et al., 2010; Miyajima et al., 2014). Human-induced pluripotent stem cell (iPSC)-derived hepatocytes (hiHeps) are genetically more closely related to PHH than are hepatoma cell lines (Gao and Liu, 2017). The hiHeps have demonstrated the capacity for bile production in sandwich culture, as shown by total BA syntheses and responses toward hepatoprotective substances (Ni et al., 2016). Regardless of the transcriptomic study conducted (Gao and Liu, 2017), hiHeps still exhibit inferior drug metabolic properties compared with HepaRG (Kvist et al., 2018). An *in vitro* experiment validated that hiHeps possess lowered CYP protein, particularly CYP7A1, which decreased the amount of total BA by 30% PHH (Ni et al., 2016). Additionally, it expressed low BSEP activities as opposed to MRP2 (Sakai et al., 2019). Thus, optimum modulation of iPSC differentiation toward liver cells is necessary, considering that hiHeps has a high potential for bile testing.

### Modulation in Tissue-Culture Method

Methods for culturing liver tissue have been known to create an ideal environment to support liver-cell physiology, including bile production. Such bile production can be sustained through the maintenance of bile canaliculi between adjacent hepatocytes where bile is first secreted. One approach maintains the oxygen supplies toward culture system to support high hepatocyte metabolism rates (Giglioni et al., 2018). Hepatocyte culture has been established on oxygen-permeable polydimethylsiloxane to maintain hepatocyte bile canaliculi. This culture model exhibits favorable morphology and function of hepatocytes over hepatocyte cultures on polystyrene surfaces (Matsui et al., 2010; Xiao et al., 2014, 2015). Additionally, continuous direct oxygenation can be achieved using a collagen Vitrigel membrane chamber (Oshikata-Miyazaki and Takezawa, 2016). Hepatocyte cultures have shown active bile-conjugate secretion into both bile canaliculi networks and extracellular solutions.

Extracellular matrices (ECMs) have been shown to maintain bile canaliculi. Sandwich configurations have been thoroughly explored to reestablish the specific transporters on the canalicular and sinusoidal membrane domains significant for bile-based analyses (Levy et al., 2015; Yang et al., 2017). They employ various kinds of ECM proteins, including collagen (Swift et al., 2010; Chatterjee et al., 2014; Keemink et al., 2015; Deharde et al., 2016; Zeigerer et al., 2017), Matrigel (Deharde et al., 2016; Sun et al., 2019), laminin (Watanabe et al., 2016), or combinations (Swift et al., 2010; Marion et al., 2012; Fukuda et al., 2014; Keemink et al., 2015; Xiao et al., 2015; Deharde et al., 2016; Ni et al., 2016; Susukida et al., 2016; Ogimura et al., 2017). Notably, distinct ECM compositions, including layering, have had diverse impacts on liver culture. The cellular arrangement and morphology of liver cells are mainly governed by underlay ECM, whereas the canalicular network and bile secretions are affected by the overlay ECM (Deharde et al., 2016). A combination of collagen underlay–Matrigel overlay appears to be the ideal sandwich mixture needed to simulate a hepatocyte architecture and functions related to bile production. These sandwich cultures can preserve the optimum bile canaliculi network and CYP1A1/2 activity for 1 week while maintaining the culture for 2 weeks (Xiao et al., 2015; Lauschke et al., 2016). This culture model is flexible and can be combined with other culture modifications, owing to its simplicity.

A self-organized three-dimensional model in a spheroid configuration successfully improved bile production and toxicity assays. Spheroids increase cell density, cell-contact polarity, and culture plasticity, including coculture modulation and ECM inclusion (Soldatow et al., 2013; Ramaiahgari et al., 2014; Fatehullah et al., 2016). Unlike sandwich configurations, liver cultures in spheroid configurations allow multiple canalicular sites, thus maintaining superior phase I and II enzyme activities (Soldatow et al., 2013; Ramaiahgari et al., 2014) with a culture age of up to 5 weeks (Bells et al., 2016; Lauschke et al., 2016). Liver spheroids have been reported to amplify drug metabolism and bile-related performances of various cell sources, including PHH (Vorrink et al., 2017), HepaRG (Sun et al., 2019), HepG2 (Tamai et al., 2013; Yamada et al., 2015), and hiHeps as liver organoids (Lauschke et al., 2016; Ni et al., 2016; Fiorotto et al., 2019). It can also enhance BSEP expression in HepaRG cells (Sun et al., 2019). A study using 56 endogenous compounds demonstrated a 3-week stability of endogenous and xenobiotic metabolites in the PHH spheroid. Notably, the BA composition excreted by the PHH spheroid contained higher glycine-conjugated BA compared with freshly isolated PHH (Vorrink et al., 2017). Cocultures with liver non-parenchymal cells, such as fibroblasts (Tamai et al., 2013; Ware et al., 2015; Underhills and Khetani, 2018), Kupfer cells, bile duct cells, and stellate cells (Bells et al., 2016; Baze et al., 2018; Underhills and Khetani, 2018) stabilize the PHH function. They also support the PHH culture for long-term exposure toxicity tests. These cocultures displayed a three-fold higher BA accumulation as a response to chlorpromazine in cholestatic presence (Bells et al., 2016).

Although liver spheroids display merits for *in vitro* liver cultures, the model has several limitations. First, the greater the size and density of the liver spheroid, the more susceptible it is to necrotic core development (Astashkina and Grainger, 2014). Only spheroids having diameters of ~200 μm (1.5–2 × 10^3^ cells/spheroids) receive adequate oxygenation that can reach the spheroid core (Bells et al., 2016; Ahmed et al., 2017). ECM incorporation of spheroid collagen fibrils (Tamai et al., 2013), collagen microparticles (Ahmed et al., 2017), encapsulations (Rebelo et al., 2015; Chan et al., 2016), and bioprinting techniques (Kizawa et al., 2017) have reportedly permitted greater oxygenation. The BA production increases two-fold on day 4 from the prior day in PHHs (Kizawa et al., 2017). Second, liver spheroids possess multiple canalicular networks, yet they serve as a close system for bile fluids collection. A micropatterned collagen gel can organize liver aggregates in their spheroid formation while sustaining their metabolic function. Notably, these spheroids have an enlarged bile canaliculi site that is openly exposed to the culture medium, enabling direct bile fluids collection. The bile canaliculus accumulates a bile analog that is successfully recovered using an oil injector. The quantity of recovered bile is 27 × greater than that of the sandwich culture (Matsui et al., 2012). Based on this study, the presence of an outlet that feasibly extends the canalicular network can realize the bile fluids collection *in vitro*.

A dynamic culture liver model employing microfluidic technology (Nakao et al., 2011; Zhou et al., 2015; Haque et al., 2016; Wang et al., 2018) has drawn attention for bile canaliculi and as a bile outlet establishment. A sinusoidal-like fluidic chamber effectively aligns hepatocytes and controls the bile canaliculi formation corresponding to the hepatic cord structure (Nakao et al., 2011). Furthermore, the microfluidic platform enhances the maturation of hiHeps organoids (Wang et al., 2018). Although it has not been demonstrated, this system can feasibly provide continuous bile fluids collected from the chamber outlet.

### Utilization of Inducing Agent for Bile Secretion and Opening of Bile Canaliculus

Manipulating the gradient concentration in a culture medium can facilitate the bile outlet from a canalicular network. A gradient difference generated by Ca^2+^/Mg^2+^ stimulates the bile canaliculi opening to release bile into the culture medium. A well-established method is the B-Clear® technology. This technology has been broadly used to calculate bile excretion and accumulation from hepatocyte sandwich cultures (Marion et al., 2012; Fukuda et al., 2014; Bachour-El Azzi et al., 2015; Ni et al., 2016; Yan et al., 2017). This method creates a Ca^2+^ or Mg^2+^ concentration difference between the hepatocyte culture and the culture medium, and the depletion disrupts the bile canaliculi tight junction. It involves the utilization of Hank's balanced salt solution as a carrier buffer of Ca^2+^/Mg^2+^. A bile fraction collected from the disrupted bile canaliculi is obtained from the accumulation difference between buffer Ca^2+^/Mg^2+^ and buffer-free Ca^2+^/Mg^2+^, as presented by the biliary excretion index (BEI). This method can also be used to assess the basolateral and canalicular efflux of bile and the substance of interest by measuring the mass difference in the absence and presence of Ca^2+^/Mg^2+^ (Swift et al., 2010; Ying et al., 2018). A long-term Ca^2+^/Mg^2+^ incubation may lead to cell toxicity and irreversible bile canaliculi disruption. Postclearance treatment using AMP-activated protein kinase activators (e.g., 2-deoxyglucose, 5-aminoimidazole-4-carboxamide-1-b-riboside, metformin, and forskolin) can induce hepatocyte tissue retention while maintaining the bile canaliculi network (Ying et al., 2018). This treatment increases the applicability of long-term hepatocyte cultures with regular bile clearance. To maximize bile secretion, bile inducers or chelates can be utilized to alter bile production. Prostaglandin E_2_ (PGE_2_) is a lipid inflammatory mediator that potentially enhances bile production. The deficiency of PGE_2_ receptor subtype 3 (EP3) and 4 (EP4) downregulates the expression of CYP7A1, resulting in inhibition of BA synthesis and hypercholesterolemia (Fu et al., 2010; Brouwer et al., 2013).

The BEI determination appears to be the most convenient approach to predicting bile secretion *in vitro*. Nonetheless, this value depends on the amount associated with hepatocytes. Furthermore, the adequacy of solutes concentration in the bile canaliculi to draw water flow, which also acts as a driving force, remains unclear. Under such conditions, drug concentrations with or without bile canaliculi must be determined to estimate the amount of bile solutes secreted into the bile canaliculi.

### Integration of Multiple Tissues for Bile Transportation

In addition to the bile accumulation in canaliculi, multiple liver tissues (e.g., bile duct) and other organs (e.g., intestine) convey bile prior to their excretion from the body. These tissues and organs are also responsible for the modification of bile components, including the transformation of primary bile into secondary bile by gut-resided microbiomes (Ridlon et al., 2014; Quinn et al., 2020). The bile duct is a liver tissue that exclusively regulates the accumulation and transportation of bile inside the liver, and it consists of intra- and extrahepatic biliary ducts (Lemaigree, 2009; Boyer, 2013; Chiang, 2013; Han et al., 2013; Matsui et al., 2018). The inclusion of a bile duct *in vitro* not only demonstrates the hepatobiliary interaction, but it also suggests bile recovery outlet from the liver culture.

Hepatobiliary organoids established from liver progenitor cells using multiple apparatuses [e.g., decellularized liver scaffolds, U-bottom plates, and coated plates (Katsuda et al., 2013; Vyas et al., 2018; Wu et al., 2019)] are promising for physiologically collecting bile from hepatocytes. A study demonstrated bile accumulation in the bile duct sac/cyst that was situated in the organoid core (Katsuda et al., 2013). Nonetheless, the location of the bile duct in this organoid makes bile fluids collection difficult. The development of bile-duct organization is potentially instrumental in resolving this hindrance. Some studies have demonstrated that the bile duct can be independently reconstructed *in vitro*. Microstructures and scaffold gels are effective for spatially controlling biliary cells to form functional bile-duct cysts (Miura et al., 2018; Rizki-Safitri et al., 2018; Funfak et al., 2019) and tubes (Du et al., 2020) using cells from rodents. These biliary structures express active bile transporters under a rich-laminin ECM environment. Laminin is essential for bile duct polarity and is thus frequently utilized for bile-duct development from iPSCs (Tanimizu et al., 2007, 2012; Kido et al., 2015; Sampaziotis et al., 2015). Attempts to integrate hepatobiliary cultures have demonstrated the transportation of bile conjugates from hepatocytes to the bile-duct structure. Liver spheroids comprising hepatocytes, biliary cells, and fibroblasts have had biliary cyst structures on their periphery. The structures developed into duct-like structures that connected liver spheroids while possibly transporting the bile conjugate (Hafiz et al., 2021). A collagen culture insert has demonstrated the likelihood of transporting bile conjugate from the hepatocyte to the bile-duct structure (Rizki-Safitri et al., 2020). The referred study showed the potential of separate autologous bile clearances suitable for long-term toxicity testing. Although bile-duct inclusion is promising for bile fluids collection *in vitro*, independent bile-duct structures are unable when demonstrating hepatobiliary bile fluids transportation. The bile-duct function has always been associated with bile canaliculus in adjacent hepatocytes. Furthermore, bile ducts *in vitro* remain immature. Thus, they are unlikely to perform optimum bile fluids collection. The technique for integrating hepatobiliary using membranes also diminishes direct hepatobiliary contact, resulting in bile leakage.

A multitissue/organ culture that incorporates liver tissue with other organs might simplify the recovery process. In addition to the blood stream, the intestine is the subsequent organ where bile is disembogued and experiences further modification (Boyer, 2013; Chiang, 2013). A microfluidic liver–intestine platform can demonstrate relations between biological processes in the liver and intestine, including processes related to bile production and secretion (Maschmeyer et al., 2015; Chen et al., 2017; Choe et al., 2017). This system allows the direct impact of bile, particularly in demonstrating interorgan drug-dependence studies. However, considering that the intestine displays two-way interactions with the liver, these intestine–liver platforms focus on drug absorption in the digestive tract instead of *vice versa*. Additionally, an integrated platform that combines organoids from six organs demonstrates the alteration of liver metabolites using human-relevant dose drug dependence. This system exhibits the activation of a prodrug into an active drug that rarely occurs in the absence of liver organoids. Analyses of the liver organoid metabolite displays the presence of 5-fluorouracyl, which is a product of capecitabine metabolism by the liver. 5-Fluorouracyl is highly toxic and destructive to heart and lung organoids as downstream organs in a microfluidic platform (Skardal et al., 2020). Little is known about the bile composition and whether this metabolite is toxic specifically toward the heart and lung or merely to adjacent tissues/organs.

## Conclusion/Outlook

Bile assays may offer numerous advantages to complement standard *in vitro* liver function analyses. Recent studies have shown that the liver-culture model enables *in vitro* bile production and collection. *In vitro* bile fluids collection can be potentially used as a supportive assay in the liver model. It can also be used to understand drug effects and secretion processes. The available bile fluids collection model exhibits flexibility toward modulations and integration with technologies, such as microfluidic devices. It allows an integrated liver tissue that is promising for recreating multitissue organization, which is advantageous for *in vitro* bile fluids collection and clearance. We know that not all types of cells or technology are desirable for bile analyses. Hence, the determination of appropriate culture modulations will increase the efficiency and appositeness of *in vitro* bile analyses. Altogether, complex liver tissue is substantial in establishing a relevant *in vitro* liver applicable for broader preclinical assays.

## Author Contributions

AR-S and YS conceived the review. AR-S, FT, and MN drafted the manuscript. AR-S and FT generated the figure and table. AR-S, FT, MN, MT, KM, HK, and YS critically revised the manuscript. All authors contributed to the article and approved the submitted version.

## Conflict of Interest

The authors declare that the research was conducted in the absence of any commercial or financial relationships that could be construed as a potential conflict of interest.
